# Circular RNA CELF1 drives immunosuppression and anti-PD1 therapy resistance in non-small cell lung cancer via the miR-491-5p/EGFR axis

**DOI:** 10.18632/aging.203576

**Published:** 2021-11-17

**Authors:** Wen Ge, Hao Chi, Hua Tang, Jianjun Xu, Jing Wang, Wan Cai, Haitao Ma

**Affiliations:** 1 Department of Cardiothoracic Surgery, Shuguang Hospital, Affiliated to Shanghai University of Traditional Chinese Medicine, Shanghai, PR China; 2 Department of Thoracic Surgery, Changzheng Hospital, Affiliated to Naval Medical University, Shanghai, PR China; 3 Department of Thoracic Surgery, The First Affiliated Hospital of Soochow University, Suzhou, PR China

**Keywords:** circ_CELF1, NSCLC, miR-491-5p, EGFR

## Abstract

Aim: To explore the immunoregulatory effects of circ_CELF1 in non-small cell lung cancer (NSCLC).

Methods: The mRNA level of circ_CELF1 in primary tissue samples was analyzed by qRT-PCR. The assays of CCK-8, colony formation, wound healing as well as Transwell were employed for measurement of cancer cell malignant transformation. The murine subcutaneous tumor model was used to assess the tumorigenesis of NSCLC *in vivo*. The assays of circRNA precipitation, RNA immunoprecipitation, and luciferase reporter were performed to study the relationship between circ_CELF1, miR-491-5p, and EGFR.

Results: circ_CELF1 is upregulated in primary cancer tissues from patients with NSCLC, and a high level of circ_CELF1, is associated with malignant characteristics and poor outcomes of patients with NSCLC. Enforced expression of circ_CELF1 exacerbated the malignant transformation of NSCLC cells. Mechanistically, through directly interacting with miR-491-5p, circ_CELF1 acted as a miRNA sponge that increased the expression of the miR-491-5p target gene EGFR, eventually promoting the progression of NSCLC and increasing cancer resistance to immunotherapy.

Conclusion: Our data demonstrate that upregulation of circ_CELF1 elicits both oncogenic and immunoregulatory effects on the development of NSCLC. We believe that circ_CELF1 can act as a potential therapeutic target for the treatment of NSCLC.

## INTRODUCTION

Non-small cell lung cancer (NSCLC), accounting for 80% of lung cancer, is one of the malignant tumors with the highest morbidity and mortality in China [1, 2]. It mainly comprises adenocarcinoma (65%) and squamous cell carcinoma (30%) histologies. In recent years, immunotherapy has greatly improved the prognosis of adenocarcinomas and squamous cell carcinomas, whereas the treatment of targetable driver mutations, has so far only benefited adenocarcinomas [1, 2]. The prognosis of patients is not only related to tumor stage and pathological type but also closely related to the regulation of tumor cell proliferation, injury repair, anti-apoptosis, and other associated genes. In addition, in NSCLC patients, visceral metastasis occurs in 50%, which has a severe impact on the patients’ physical and mental health, quality of life, and prognosis. Surgery belongs to the traditional treatment of NSCLC, but there are no typical symptoms in the early stage of NSCLC [3, 4]. The clinical signs are not specific, so the vast majority of patients have been confirmed to be late stage at the time of diagnosis and are not suitable for surgical treatment. Although significant progress has been made in the treatment methods, treatment equipment, and technology of lung cancer at this stage, the outcome of patients with NSCLC is still relatively poor. We need to further explore more markers for clinical diagnosis and therapy of NSCLC.

As a new type of non-coding RNA, circular RNAs (circRNAs) with covalently closed ring structures were once considered as a shear intermediate, a by-product of shear, or a product of mis-cut [5, 6]. Intron pairing-driven cyclization and lasso-driven cyclization are the main pathways of circRNAs synthesis. CircRNAs could adsorb miRNA, regulate the expression of target mRNA by miRNA, and play a regulatory role by binding to proteins or translating into short peptides [7]. For instance, circ_HIPK3 can bind to a variety of miRNA through the action of the miRNA sponge, thus regulating cell growth [8]. Besides, Hang et al. found that the level of circ_FARSA in plasma from patients with NSCLC was significantly increased. Circ_FARSA weakens the inhibitory effect of microRNAs on oncogene fatty acid synthetase by adsorbing miR-3330-5p and miR-326, while overexpressed circ_FARSA in NSCLC cell line A549 significantly promotes tumor cell proliferation and migration [9]. Similar results were also detected by Zhang et al., who found that circ_FGFR1 was also upregulated in NSCLC tissues, which was closely related to poor prognoses of patients with NSCLC. Through binding with miR-381-3p, circ_FGFR1 decreased the expression of CXCR4, which is the target gene of miR-381-3p, and exacerbated the progression of NSCLC [10]. At present, there are few studies on NSCLC-related circRNAs. It can be predicted that with the in-depth analysis of the molecular mechanism of circRNAs, more and more NSCLC-related circRNAs would be found. Circ_CELF1 is generated from backsplicing of CELF1 gene. CELF1, also known as CUG binding protein 1 (CUBP1), is a multifunctional RNA binding protein, that binds to GU rich elements in 3′UTR of target RNAs to regulate their stability [11]. CELF1 expression is found to increase proliferation and progression of several cancers, whereas increased CELF1 could cause G1 phase growth arrest in intestinal epithelial cells, suggesting its diverse role in carcinogenesis [12, 13]. Here, we wonder if circ_CELF1 expression is correlated with NSCLC progression.

Epidermal growth factor (EGF) signal pathway, including EGFR and HER-1, plays a crucial role in the occurrence and progression of tumors [14, 15]. A high level of EGFR is associated with poor sensitivity to radiotherapy and chemotherapy and poor prognosis [16]. Activation of EGFR inhibits the apoptotic system in tumor cells and promotes tumor cell proliferation, angiogenesis and metastasis.

Here, we found that circ_CELF1 was significantly upregulated in NSCLC tumor tissues, which was positively correlated with the poor prognosis of patients with NSCLC. Silencing of circ_CELF1 inhibited NSCLC progression by sequestering miR-491-5p and remitted the expression of EGFR. Our research identified that circ_CELF1 functions as a promoter in NSCLC progression and may be a potential target in NSCLC prediction and therapy.

## RESULTS

### Circ_CELF1 is upregulated in primary cancer tissues of NSCLC

**Figure 1:**
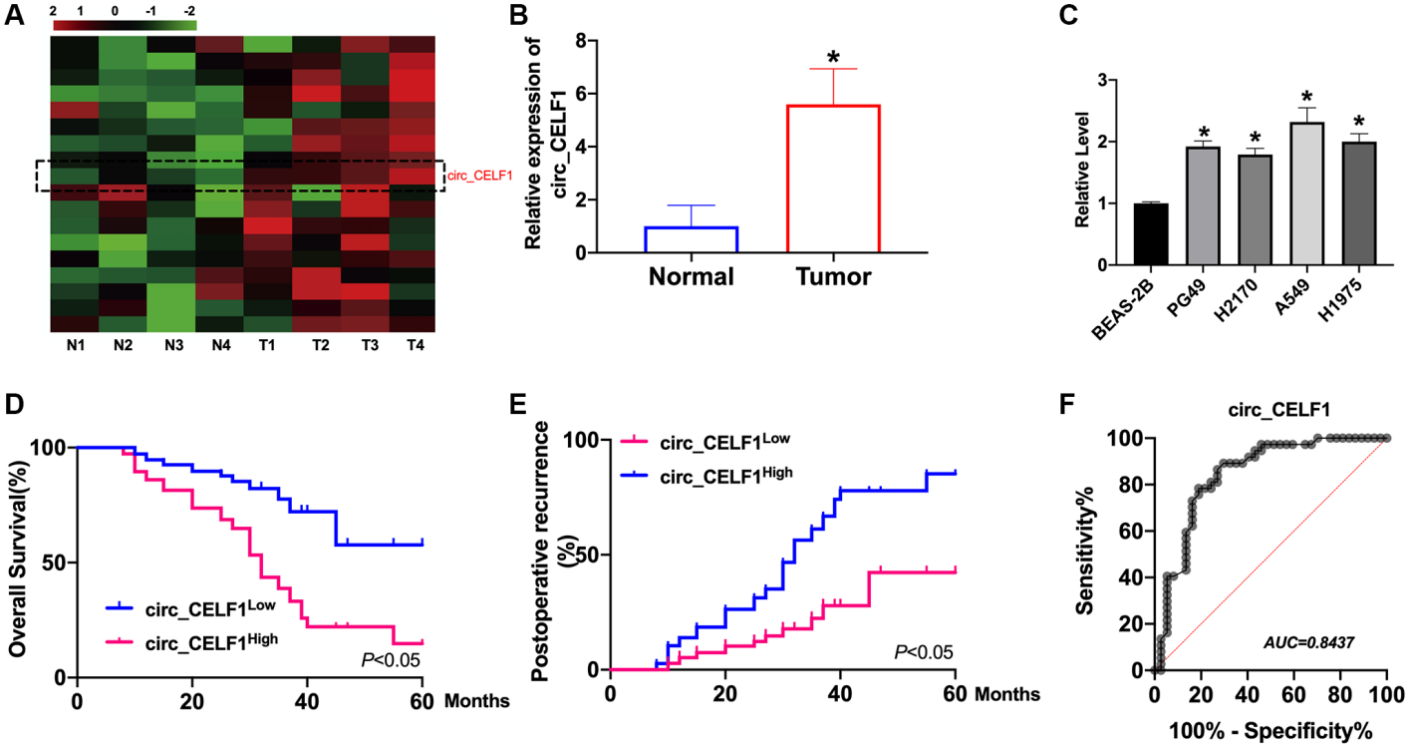
**The high circ_CELF1 expression in NSCLC tissues and prognostic significance.** (**A**) The heatmap shows circRNAs derived from the CELF1 gene in NSCLC tissues compared with those in adjacent normal tissues analyzed by qRT-PCR. (**B**) The differential expression of circ_CELF1 in NSCLC tissues and adjacent normal tissues NSCLC patients was determined by qRT-PCR. *n* = 37, ^*^P < 0.05. (**C**) The expression level of circ_CELF1 in NSCLC cell lines (PG49, H2170, A549, H1975) was detected by qRT-PCR assay, and BEAS-2B was indicated as control. *n* = 5, ^*^P < 0.05. (**D** and **E**) Survival (**D**) and recurrence (**E**) analysis of circ_CELF1 high and low patients in 30 NSCLC patients. (**F**) ROC curve analysis was performed to evaluate the diagnostic value of circ_CELF1 in NSCLC by using 37 primary NSCLC tissues.

Dysregulation of circRNAs is involved in tumor progression by different mechanisms. We first identified circ_CELF1 as circular RNA. We observed that circ_CELF1 was detectable under the treatment of RNase R and the linear form of CELF1 was not observed using convergent primer (Supplementary Figure 1A). Moreover, the half-life of circ_CELF1 under actinomycin D treatment was notably longer than that of linear CELF1 (Supplementary Figure 1B). These data together identified the stability of circ_CELF1 as a circular RNA. Moreover, FISH assay showed the localization of circ_CELF1 in cytoplasm fraction, confirming the feature of circ_CELF1, and its possible role as a miRNA sponge (Supplementary Figure 1C). To investigate the status of circ_CELF1 in the development of NSCLC, we employed qRT-PCR and found that the mRNA level of circ_CELF1 was significantly increased in primary cancer tissues as relative to their adjacent normal tissues (Figure 1A and 1B). Higher level of circ_CELF1 was spotted in NSCLC cell lines, comparing with the normal human epithelial cells (Figure 1C). To study the relationship of circ_CELF1 upregulation with the outcome of patients with NSCLC, we performed Kaplan-Meier survival analysis. As shown in Figure 1D, high level of circ_CELF1 expression is correlated with the poor prognosis of patients with NSCLC. Besides, Higher level of circ_CELF1 is notably correlated with elevated recurrence of patients after surgical operation (Figure 1E). Diagnostic performance of circ_CELF1 was determined by the calculated sensitivity, specificity, and area under the receiver operating characteristic curve (AUC). ROC curve showed that circ_CELF1 level in tissues can discriminate NSCLC tissues with a sensitivity of 89.7% and specificity of 90.9% from non-cancer control (Figure 1F).

### Enforced expression of circ_CELF1 drives tumor progression *in vitro*

**Figure 2:**
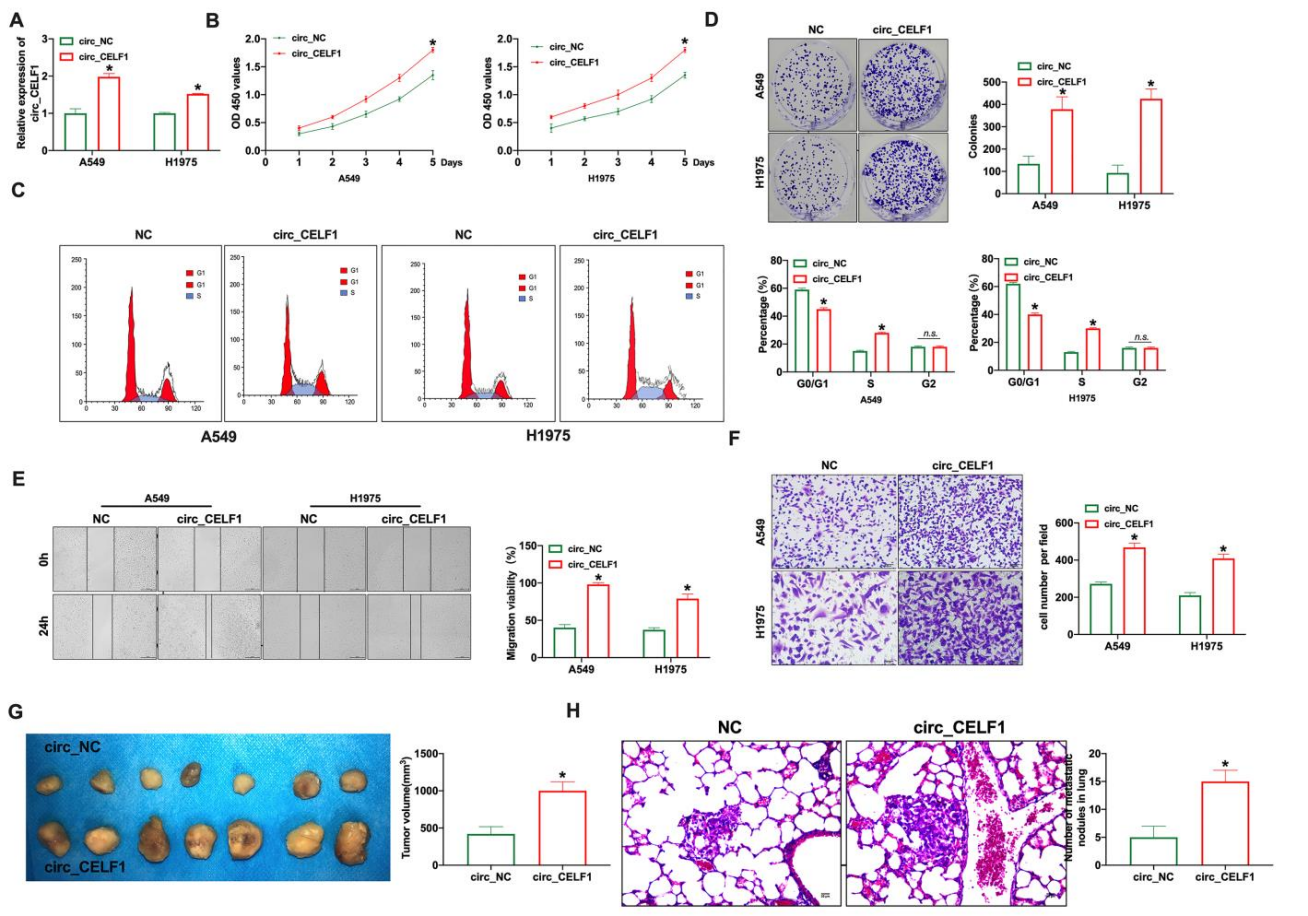
**Forced expression of circ_CELF1 induces the progression of NSCLC cells *in vitro* and *in vivo*.** (**A**) The qRT-PCR was performed to confirm the transfection efficiency of circ_CELF1 in NSCLC cells. *n* = 5, ^*^P < 0.05. (**B**) CCK-8 assay was performed to detect the cell viability on A549 and H1975 cells. *n* = 6, ^*^P < 0.05. (**C**) The cell cycle was analyzed on A549 and H1975 cells. *n* = 3, ^*^P < 0.05. (**D**) The clone formation assay on A549 and H1975 cells. *n* = 3, ^*^P < 0.05. (**E**) The wound healing assay was performed on A549 and H1975 cells. *n* = 3, ^*^P < 0.05. (**F**) The ability of invasion was explored on A549 and H1975 cells by Transwell. *n* = 4, ^*^P < 0.05. (**G**) Tumor growth in NSCLC cells with the forced expression of circ_CELF1 was investigated by xenograft tumor models. *n* = 7, ^*^P < 0.05. (**H**) The number of metastatic nodules of the lung was counted in two groups. *n* = 6, ^*^P < 0.05.

To study the biological function of circ_CELF1 in NSCLC, we constructed the vector of circ_CELF1. As shown in Figure 2A, the transfection efficiency of circ_CELF1 1 was detected in lung cancer cell lines (A549 and H1975). Through CCK-8 assay, enforced expression of circ_CELF1 promotes the proliferation of A549 and H1975 cells (Figure 2B). We next used flow cytometry to analyze the cell-cycle distribution of NSCLC cells. As shown in Figure 2C, enforced expression of circ_CELF1 triggered cell cycle progression from G0/G1 to S phase. In accordance with the cell cycle data, overexpression of circ_CELF1 promoted colony formation in both A549 and H1975 cells (Figure 2D). Noteworthy, the transfection of negative control vector did not change the level of circ_CELF1, as well as not affect cell proliferation, migration and invasion (Supplementary Figure 2).

To determine whether circ_CELF1 also affects the ability of migration and invasion, we used wound-healing and transwell assays. As shown in Figure 2E and 2F, circ_CELF1 promoted the migration of both A549 and H1975 cells. The promoting role of circ_CELF1 was also confirmed in mouse NSCLC cell line LCC and LAA795 (Supplementary Figure 3). Next, Xenograft assay was carried out to explore the tumor progression. circ_CELF1-expressing or circ_NC-expressing A549 cells were subcutaneously injected to the left hind limb of the mouse constructed the tumor-bearing mode. The results showed that circ_CELF1 induced tumor progression *in vivo* (Figure 2G). We then detected pulmonary metastasis by vein injection and found that circ_CELF1 influenced pulmonary nodules upregulation (Figure 2H). Taken together, enforced expression circ_CELF1 promotes tumor progression *in vivo* and *in vitro*.

### Circ_CELF1 directly binds to and limits biological function of miR-491-5p

**Figure 3:**
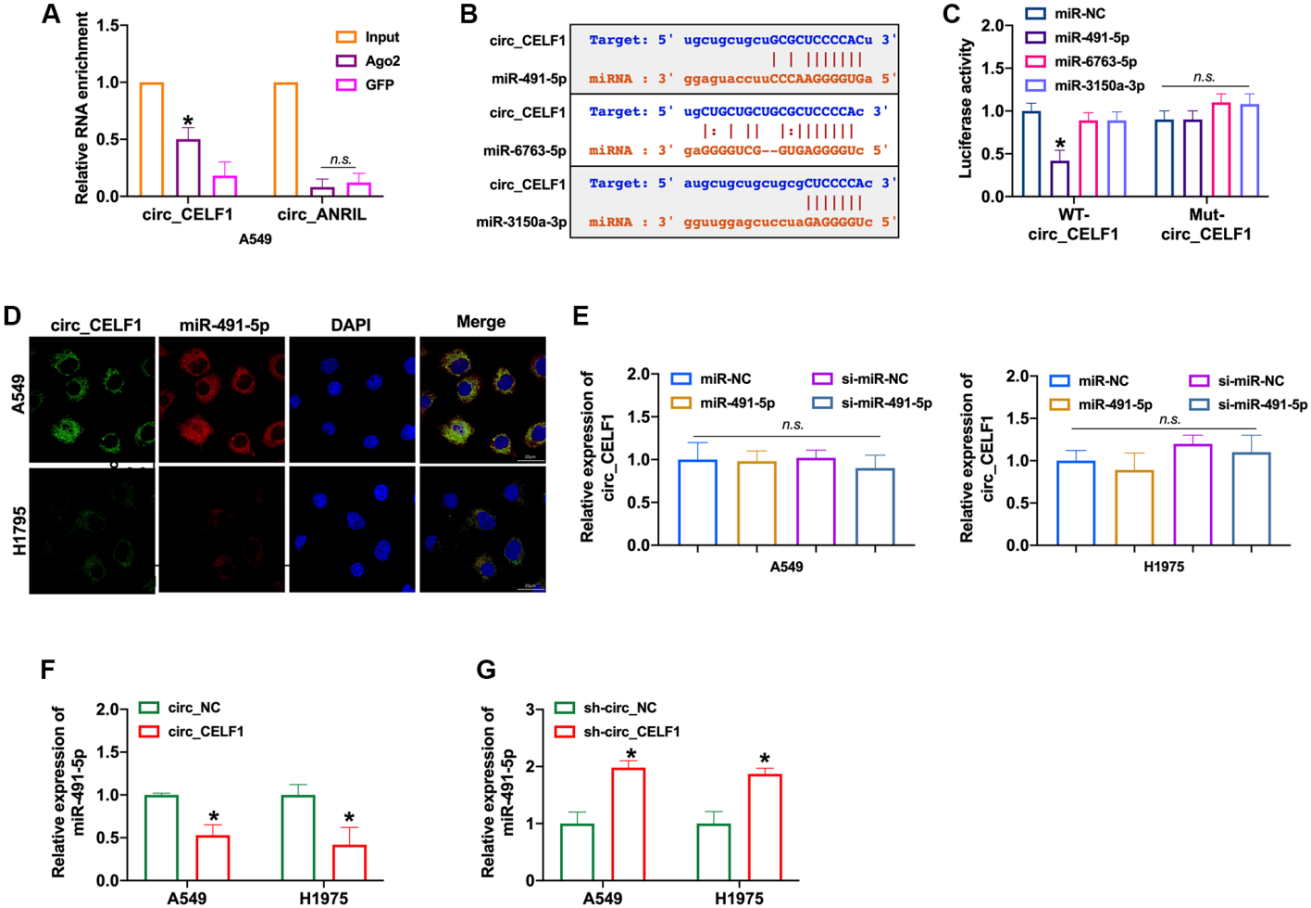
**circ_CELF1 binds miR-491-5p in NSCLC cells.** (**A**) AGO2 RIP experiments were performed using an antibody against Ago2 on extracts from A549 cells. *n* = 3, ^*^P < 0.05. (**B**) A schematic drawing showing the putative binding sites of miR-491-5p, miR-6763-5p, and miR-3150a-3p with respect to circ_CELF1. (**C**) The luciferase activity of WT- circ_CELF1 or mutant circ_CELF1 in HEK293 cells after co-transfection with miR-491-5p, miR-6763-5p, and miR-3150a-3p. *n* = 3, ^*^P < 0.05. (**D**) A549 and H1975 cells were transfected with miR-491-5p or miR-NC for 48 h. The association between circ_CELF1 and miR-491-5p was assessed by FISH assay. (**E**) The expression of circ_CELF1 was detected by qRT-PCR under transfection of miR-491-5p mimic and si-miR-491-5p. *n* = 4, ^*^P < 0.05. (**F**–**G**) The expression of miR-491-5p was explored by upregulation or downregulation of circ_CELF1. *n* = 4^, *^P < 0.05.

CircRNAs could bind with miRNAs through the competitive endogenous RNA (ceRNA) mechanism. RIP with AGO2 is a classic experiment to determine the role of circRNA as sponge of miRNA, basing on the direct interaction between AGO2 protein and miRNA during its splicing. As RIP assay shown, circ_CELF1 rather than circ_ANRIL was significantly enriched by anti-AGO2 (Figure 3A and Supplementary Figure 4A). The immunoprecipitation of circ_CELF1 by AGO2 antibody hence indicated the binding of circ_CELF1 to miRNAs, suggesting its role as ceRNA. Moreover, the bioinformatics analysis predicted that circ_CELF1 could bind to miR-491-5p, miR-6763-5p as well as miR-3150a-3p (Figure 3B and Supplementary Figure 4B). Then luciferase assay showed altered activity of circ_CELF1 reporter gene vector under transfection of miR-491-5p rather than miR-6763-5p or miR-3150a-3p, verified the association of circ_CELF1 with miR-491-5p (Figure 3C and Supplementary Figure 4C). FISH assay also confirmed the relationship between circ_CELF1 and miR-491-5p, manifested by their co-localization (Figure 3D). To determine whether miR-491-5p affects the expression of circ_ CELF1 in A549 cells, we transfected miR-491-5p into A549 cells. As shown in Figure 3E, miR-491-5p elicited little effect on the expression of circ_CELF1. While circ_CELF1inhibited the expression of miR-491-5p, silencing of circ_CELF1 induced the expression level of miR-491-5p (Figure 3F and 3G, and Supplementary Figure 4D). Our data thus demonstrate that circ_CELF1 sponges miR-491-5p and restricts the expression of miR-491-5p.

### MiR-491-5p limited the progression of cancer with circ_CELF1 expression

**Figure 4:**
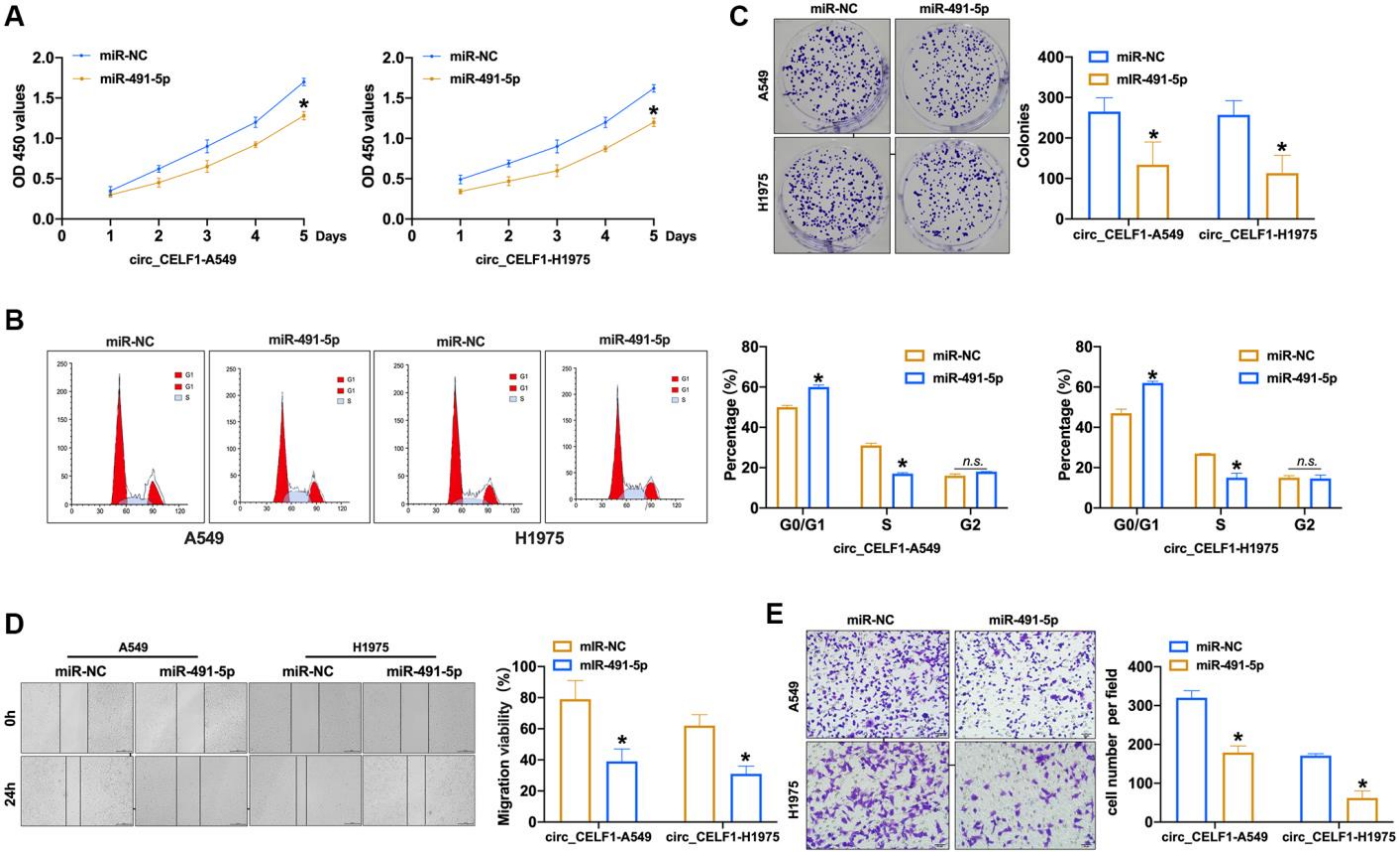
**Forced expression of miR-491-5p remits the cell progression in stabled circ_CELF1 expression NSCLC cells.** (**A**) CCK-8 assay was performed to detect the effect of miR-491-5p on overexpression of circ_CELF1 A549 and H1975 cells. *n* = 6, ^*^P < 0.05. (**B**) The cell cycle was explored by flow cytometry on overexpression of circ_CELF1 A549 and H1975 cells after miR-491-5p transfection. *n* = 6, ^*^P < 0.05. (**C**) The clone formation assay was performed on overexpression of circ_CELF1 A549 and H1975 cells. *n* = 5, ^*^P < 0.05. (**D**) Wound healing assay was used to confirm the migration ability. *n* = 5, ^*^P < 0.05. (**E**) The migration and invasion ability was explored by Transwell. *n* = 6, ^*^P < 0.05, ^**^P < 0.01.

To explore the effects of miR-491-5p on the progression of cancer with high level of circ_CELF1, we transfected miR-491-5p into circ_CELF1-expressing NSCLC cells. As shown in Figure 4A to 4B and Supplementary Figure 5A to 5B, enforced expression of miR-491-5p inhibited the proliferation of circ_CELF1-expressing human and mouse lung cancer cells through induction of cell cycle arrest. Furthermore, miR-491-5p suppressed the colony formation of circ_CELF1-expressing cells (Figure 4C and Supplementary Figure 5C). Besides, miR-491-5p remitted cell migration and invasion in circ_CELF1-expressing A549 and H1975 cells (Figure 4D and 4E, and Supplementary Figure 5D and 5E). Taken together, miR-491-5p attenuates tumorigenesis induced by circ_CELF1. In summary, miR-491-5p could involve in circ_CELF1 regulating tumor progression.

### EGFR is the downstream target of miR-491-5p

**Figure 5:**
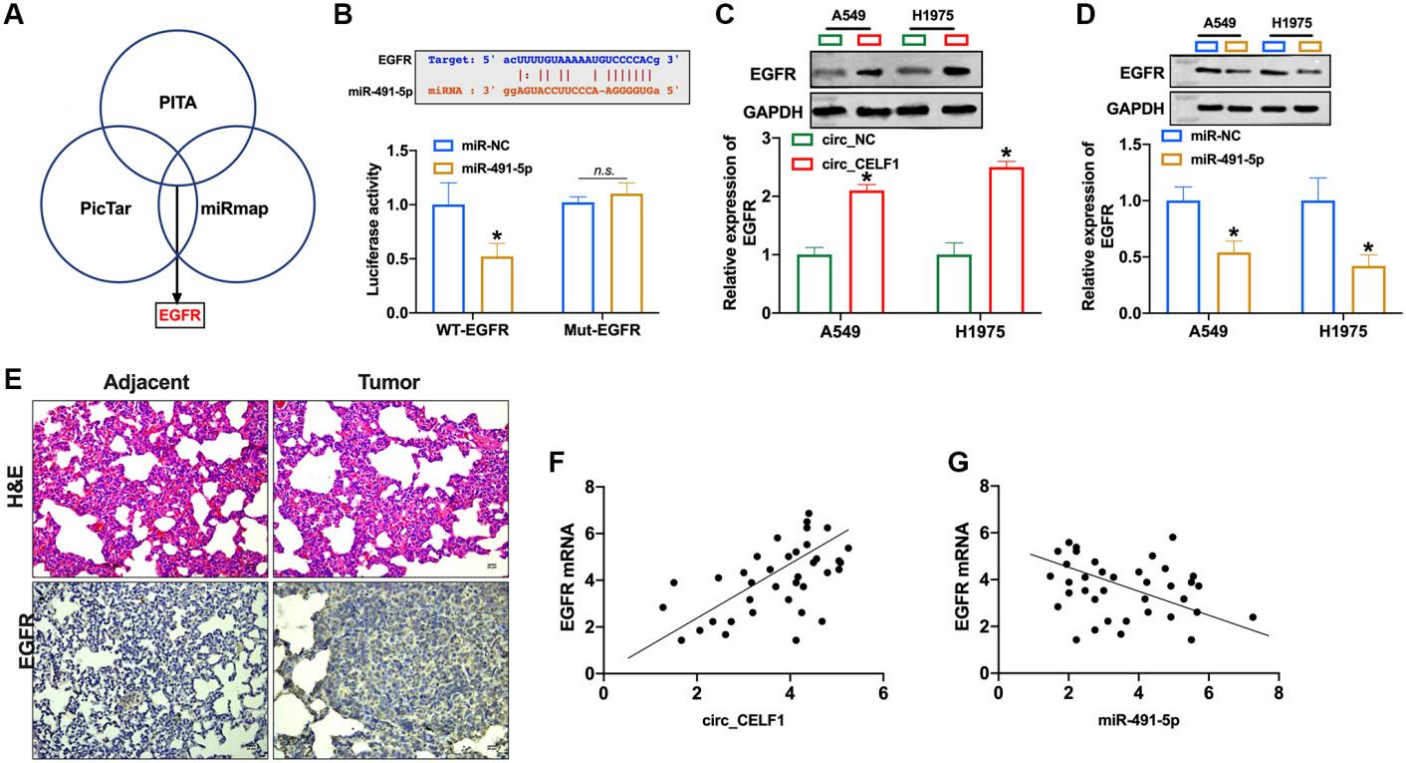
**EGFR is a target of miR-491-5p.** (**A**) Three bioinformatics sites predict the target of miR-491-5p. (**B**) The prediction binding sites between EGFR and miR-491-5p (upper). Luciferase assay confirmed the relationship between EGFR and miR-491-5p (lower). *n* = 3, ^*^P < 0.05. (**C** and **D**) The expression of EGFR was detected by qRT-PCR in A549 and H1975 cells after circ_CELF1/miR-491-5p transfection. *n* = 3, ^*^P < 0.05. (**E**) H&E staining and EGFR staining in tumor tissue and matched adjacent normal tissues. (**F**) The expression of EGFR in 37 cases of NSCLC tissues was detected by qRT-PCR. A positive correlation between circ_CELF1 and EGFR was observed in tumor tissues at the mRNA levels. *n* = 37, ^*^P < 0.05. (**G**) A negative correlation between miR-491-5p and EGFR was observed in tumor tissues at the mRNA levels. *n* = 37, ^*^P < 0.05.

Activation of EGFR signaling is involved in the occurrence and development of NSCLC. Four bioinformatics sites predicted that EGFR was the target of miR-491-5p (PITA, miRmap, and PicTar, Figure 5A), and the prediction binding sites was shown in Figure 5B. In contrast to mutant-EGFR (Mut-EGFR), transfection of WT-EGFR decreased luciferase activity of cells in presence of miR-491-5p (Figure 5B and Supplementary Figure 6A). Then we found that overexpression of circ_CELF1 induced EGFR expression in A549 and H1975 cells (Figure 5C and Supplementary Figure 6B). The expression of EGFR was decreased in NSCLC cells after miR-491-5p transfection (Figure 5D and Supplementary Figure 6C). To further confirm this result, we employed 37 NSCLC patient tissues as well as their adjacent normal tissues. As shown in Figure 5E, high level of EGFR was detected in tumor tissue from NSCLC patient. Moreover, a positive relationship between circ_CELF1 and EGFR was observed in NSCLC patients (Figure 5F), and negative relationship between miR-491-5p and EGFR was found in NSCLC patients (Figure 5G).

**Figure 6:**
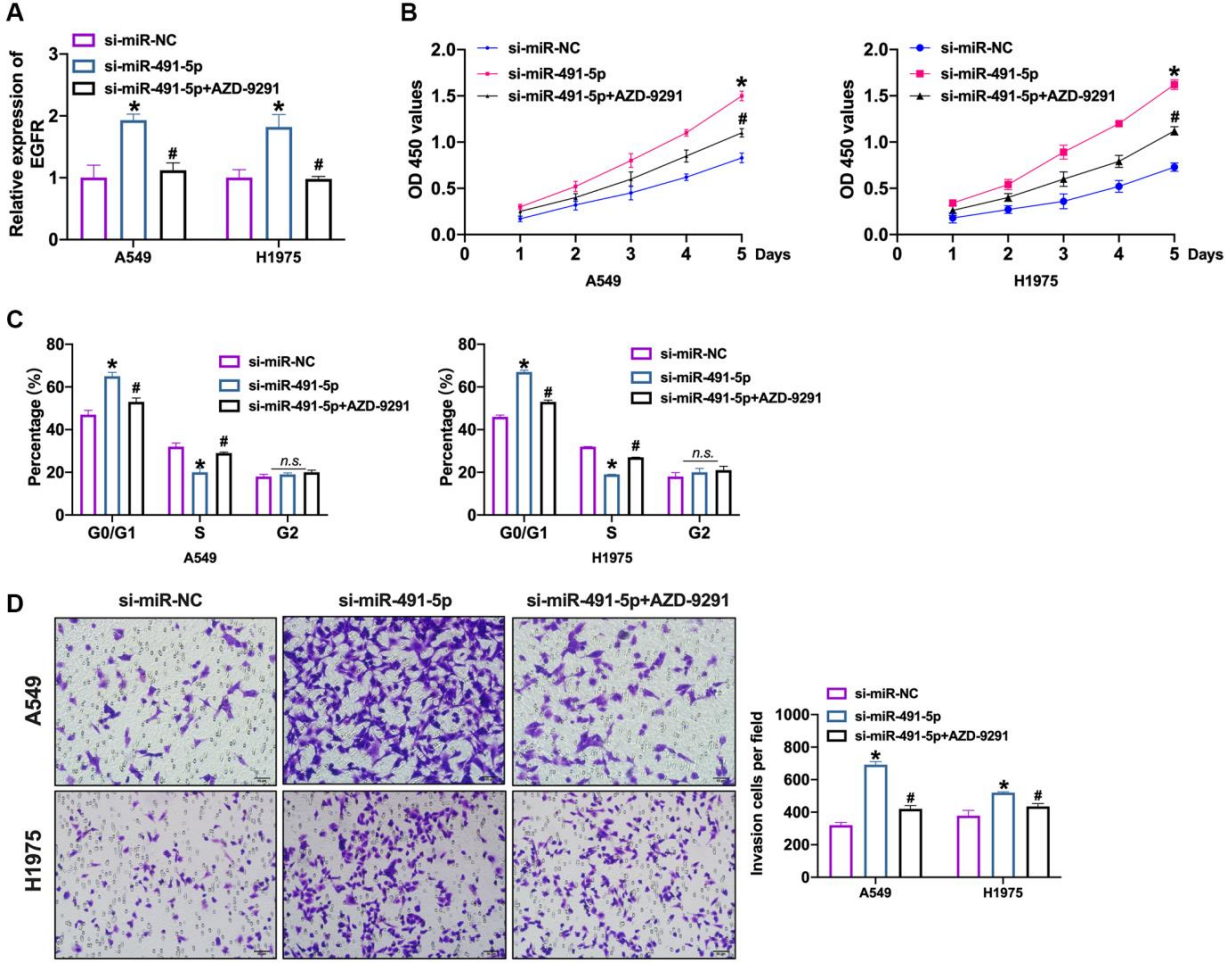
**EGFR blockage prevents NSCLC cell progression induced by miR-491-5p knockdown.** (**A**) The expression level of EGFR was confirmed by qRT-PCR. *n* = 4, ^*^P < 0.05 vs. si-miR-NC, ^#^*P* < 0.05 vs. si-miR-491-5p. (**B**) CCK-8 assay was performed to detect cell viability in NSCLC cells. *n* = 4, ^*^P < 0.05 vs. si-miR-NC, ^#^*P* < 0.05 vs. si-miR-491-5p. (**C**) The cell cycle was explored by flow cytometry in NSCLC cells after si-miR-491-5p transfection and AZD-9291 treatment. *n* = 4, ^*^P < 0.05 vs. si-miR-NC, ^#^*P* < 0.05 vs. si-miR-491-5p. (**D**) The invasion ability was explored by Transwell. *n* = 4, ^*^P < 0.05 vs. si-miR-NC, ^#^*P* < 0.05 vs. si-miR-491-5p.

Further, we transfected si-miR-491-5p/si-miR-NC into NSCLC cells after AZD-9291 treatment. We observed that silencing of miR-491-5p induced the expression of EGFR which was inverted by AZD-9291 (Figure 6A and Supplementary Figure 7A). Knockdown of miR-491-5p prevented NSCLC cells viability which was abolished by AZD-9291 (Figure 6B and Supplementary Figure 7B). Meanwhile, we performed cell cycle assay to detect the proliferation ability in NSCLC cells. Si-miR-491-5p prevented cells from G0/G1 phase into S phase, which was blocked by AZD-9291 (Figure 6C and Supplementary Figure 7C). The invasion ability was controlled by si-miR-491-5p which was recovered by AZD-9291 (Figure 6D and Supplementary Figure 7D).

### Inhibition of EGFR attenuates tumor progression in circ_CELF1 expressed NSCLC cells

**Figure 7:**
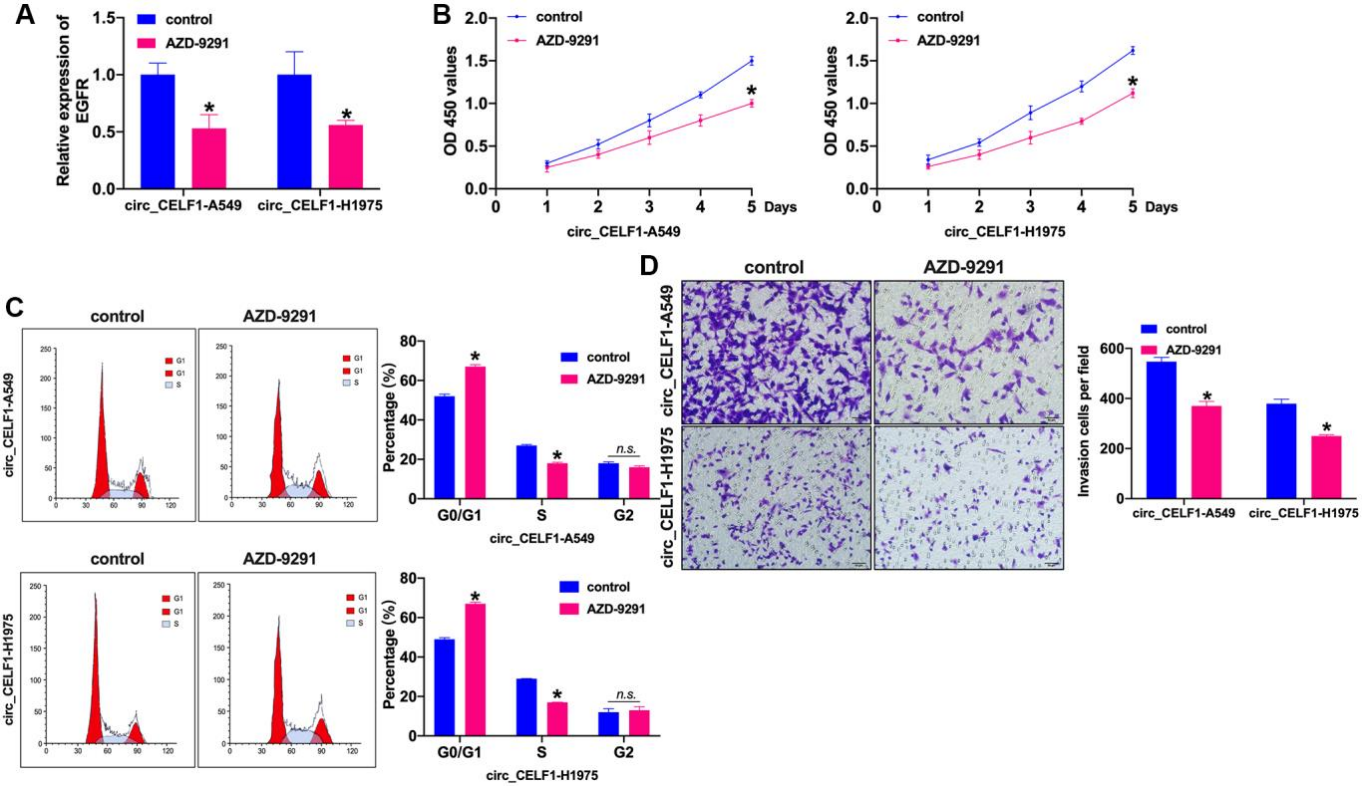
**Inhibition of EGFR prevents cell progression in stabled circ_CELF1 expressed NSCLC cells.** (**A**) The knockdown efficiency of AZD-9291 was confirmed by qRT-PCR. *n* = 4, ^*^P < 0.05. (**B**) CCK-8 assay was performed to detect the effect of MK-1775 in NSCLC cells. *n* = 4, ^*^P < 0.05. (**C**) The cell cycle was explored by flow cytometry in NSCLC cells after miR-491-5p transfection. *n* = 4, ^*^P < 0.05. (**D**) The invasion ability was explored by Transwell. *n* = 4, ^*^P < 0.05.

Whether EGFR influenced the NSCLC progression need to be confirm. AZD-9291, also named as osimertinib, is a third-generation EGFR-TKI that selectively inhibits EGFR-TKI-sensitizing and EGFR T790M mutated NSCLC, as well as exhibits obvious therapeutic efficacy and low incidence of side effects. In this work, we treated the stabled circ_CELF1 expressed cells with AZD-9291 (10 μM) for 72 h to evaluate the role of EGFR in circ_CELF1-regulated cell function. The inhibition efficiency of AZD-9291 was detected by RT-PCR (Figure 7A and Supplementary Figure 8A). Cell viability was prevented by AZD-9291 in stabled circ_CELF1 expressed NSCLC cells (Figure 7B and Supplementary Figure 8B). Meanwhile, inhibition of EGFR blocked the cell cycle from G0/G1 into S phase (Figure 7C and Supplementary Figure 8C). The stabled circ_CELF1 expressed NSCLC cells treated with AZD-9291 remitted the migration and invasion ability (Figure 7D and Supplementary Figure 8D). Taken together, EGFR participated in circ_CELF1 regulating NSCLC progression by preventing proliferation and invasion.

### circ_CELF1 limits the recruitment of T cells and promotes cancer resistance to immunotherapy

**Figure 8:**
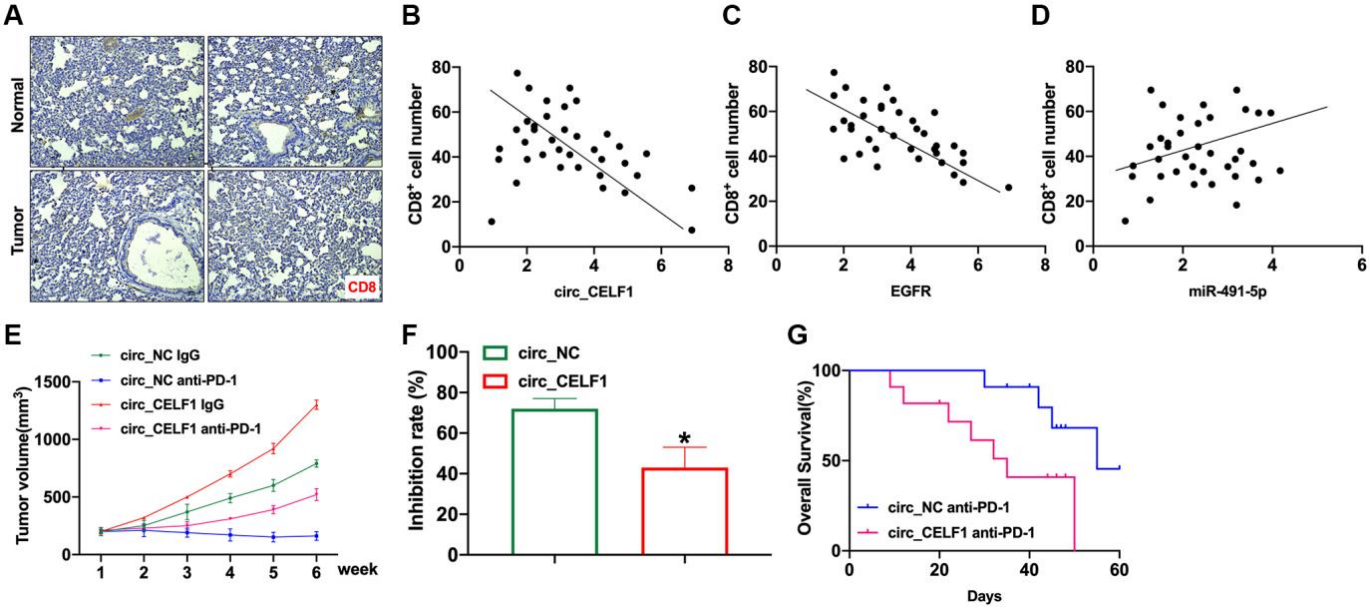
**Circ_CELF1 promotes immunosuppression and resistance to NSCLC immunotherapy.** Mouse LLC cells overexpressing circ_CELF1 was subcutaneously injected in mice to establish xenograft mice model, and evaluated immune response. (**A**) Representative NSCLC cases in which the tissue was analyzed by IHC staining for CD8. (**B**) A negative correlation between circ_CELF1 and the number of CD8+positive cells was observed in the NSCLC tissues. (**C**) A negative correlation between EGFR and the number of CD8+positive cells was observed in the NSCLC tissues. (**D**) A positive correlation between miR-381-3p and the number of CD8-positive cells was observed in the NSCLC tissues. (**E**) Circ_NC or circ_CELF1 cells were subcutaneously injected into nude mice, and when tumors reached a mean tumor volume of 100 mm^3^, the mice were treated with an IgG or PD-1 antibody. (**F**) The data are expressed as the percent of tumors with inhibited growth. (**G**) Comparison of the overall survival curves for mice with high and low circ_CELF1 expression of xenograft lung tumors that were treated with a mouse antibody against mouse PD-1.

In the past decade, immunotherapy has become mainstream research area for cancer therapy, and effectively improved the prognosis of lung adenocarcinomas. Therefore, we wonder if expression of circ_CELF1 affects immune response. As the primary executor of tumor immunity, T cells are regulated by both costimulatory molecules and inhibitory molecules. As one of the main inhibitory molecules, programmed death receptor-1 (PD-1) can inhibit T cell function through priming with PD-L1, thus facilitating cancer immune escape. We first analyzed the infiltration of CD8^+^ T cells in tissues from NSCLC and matched adjacent tissues. In accordance with previous reports, the decreased number of CD8^+^ T cells in the cancer tissues was detected as relative to that of their matched adjacent nontumor tissues (Figure 8A). To determine whether circ_CELF1 affects host antitumor immunity, we used scatter plot analysis. As shown in Figure 8B and 8C, the expression of circ_CELF1/EGFR was negatively correlated with CD8^+^ T cell frequency in the NSCLC tissues. Moreover, a positive correlation between miR-491-5p expression and CD8^+^ T cell frequency was observed (Figure 8D).

To explore the effects of circ_CELF1 expression on cancer resistance to immunotherapy, we assessed the anti-tumor effects of the PD-1 antibody in C57BL/6 mice bearing circ_CELF1-expressing or circ_NC-expressing cells. Mouse LLC cells overexpressing circ_CELF1 was subcutaneously injected in mice. As shown in Figure 8E and 8F, overexpression of circ_CELF1 impaired the tumor-suppressive effects induced by anti-PD-1 therapy, and a shorter survival time was observed in mice bearing circ_CELF1-expressing cells than mice with circ_NC-expressing cells (Figure 8G).

## DISCUSSION

At present, it has been found that most circRNAs carry at least one miRNA binding site. They can act as miRNA sponges to isolate and functionally inactivate miRNA, thereby regulating the expression of downstream target genes that miRNA suppresses by competitive endogenous RNA [17]. The most substantial evidence of sponge activity comes from the study of ciRS-7, which is a highly conservative single exon, which can regulate miR-7, by adsorption to reduce the inhibition of miR-7 on its target. Overexpression of CiRS-7 can indirectly enhance the expression of RGFR in the downstream target of miR-7, thus affecting cell growth, proliferation, differentiation and signal transduction. Like most tumors, lung cancer also shows malignant proliferation, resistance to apoptosis, enhanced invasion and unavoidable drug resistance [18]. CircPVT1 is overexpressed in tissue and serum of patients with NSCLC. Knockout of circPVT1 can inhibit cell proliferation through induction of cell cycle arrest. Pathway report chip detection found that the most significant inhibition of E2F pathway occurred after knockout of circPVT1, and overexpression of E2F2 could reverse the inhibition of cell survival after circPVT1 silencing. It is suggested that circPVT1 is expected to be developed as a target for NSCLC therapy [19]. Yu et al. identified the upregulation of circHIPK3 in lung cancer, and silencing of circHIPK3 significantly elicited stimulatory effects on apoptosis lung cancer cells and suppressed tumor growth *in vitro*. Silencing circHIPK3 can inhibit the expression of SphK1, STAT3 and CDK4, which are the potent target of miR-124. Conversely, inhibition of miR-124 can increase the expression of these target genes, suggesting that circHIPK3 transmits apoptosis resistance signals through the miR-124-SphK1/STAT3/CDK4 signal axis, thus promoting the malignant progression of lung cancer, which provides a new strategy for the treatment of NSCLC [20]. The expression of CircUBAP2 and hsa_circ_0000064 increased in lung cancer tissues. Silencing these two kinds of circRNA could inhibit cell proliferation and promote apoptosis. Silence circUBAP2 can suppress the expression of Bcl-2 and survivin.

In addition to the prolonged survival of cancer, the distant outgrowth metastasis is also related to malignant transformation of cancer. Epithelial-mesenchymal transition (EMT) is considered as the leading cause for cancer metastasis. At present, it has been found that the overexpression of TGF- β in NSCLC can stimulate the EMT of NSCLC cells. TIF1γ is down-expressed in NSCLC. Knockout or down-regulation of TIF1γ can enhance the effect of EMT induced by TGF-β. Wang first induced EMT by overexpression of TGF- β in cells, then analyzed by circRNA chip, and found that the expression of TIF1γ decreased. Therefore, it is assumed that dysregulation of circRNA impairs the expression of TIF1γ, which in turn activates EMT induced by TGF-β. According to the analysis of TargetScan/miRBase and miRanda database, it is found that both circPTK2 and TIF1γ possessed the miR-429/miR-200b-3p binding sites. Luciferase reporter gene system proved that both circPTK2 and TIF1γ directly bound to miR-429/miR-200b-3p. This study found that circPTK2 could regulate the transfer of NSCLC by inhibiting the expression of TIF1γ by sponge miR-429/miR-200b-3p, which enriches the mechanism of EMT induced by TGF-β. Qu et al. found that the expression of hsa_circ_0020123 was increased in NSCLC, and significantly correlated with lymph node metastasis and TNM stage. Knockout of hsa_circ_0020123 restricted tumor invasion and metastasis, while overexpression of hsa_circ_0020123 showed the opposite phenotype. Database prediction and luciferase reporter gene system proved that hsa_circ_0020123 can promote tumor metastasis through the miR-144-ZEB1/EZH2 axis. Here, we screened the differential expression circRNAs from NSCLC tumor tissue and matched adjacent tissues, circ_CELF1 was found significantly upregulation in tumor tissues. Forced expression of circ_CELF1 promote NSCLC progression *in vivo* and *in vitro*. Further, we found miR-491-5p could be a target of circ_CELF1. EGFR was confirmed as the downstream of circ_CELF1/miR-491-5p and involved in NSCLC tumor progression.

PD-1 and its ligand PD-L1 are negative costimulatory signal molecules newly discovered in recent years, which are mainly expressed in mature T lymphocytes and can inhibit the proliferation and activation of T lymphocytes and negatively regulate the immune response mediated by T cells [21]. PD-L1 is overexpressed in a variety of tumors, with an expression rate of 35% to 95% in NSCLC. Presence of PD-L1 in normal tissues contributes to the homeostasis of host immunity. Downregulation of PD-1/PD-L1 signaling leads to autoimmune disorders, while increased expression leads to cancer immune escape and facilitates tumor development [22]. In general, a high level of PD-L1 on tumor cells is positively correlated with the efficacy of immune checkpoint blockade therapy. However, some patients with negative PD-L1 expression also respond to anti-PD-1 antibody treatment [23]. In our research, we revealed that EGFR expression is positively regulated by circ_CELF1. Furthermore, forced expression of circ_CELF1 desensitized NSCLC tumor to anti-PD-1 immunotherapy (Supplementary Figure 9).

We discussed the biological function of circRNA and its role in tumor chemotherapy. It is helpful for us to formulate better strategies for the study of the mechanism of tumor drug resistance and to more accurately understand the important regulatory factors of therapeutic targets.

## CONCLUSIONS

In conclusion, our research demonstrates that the mRNA level of circ_CELF1 is selectively increased in NSCLC tissues as relative to their adjacent normal tissues. Enforced expression of circ_CELF1 induces the progression of NSCLC cells by sponging miR-491-5p and promoting the expression of EGFR, which is the target of miR-491-5p. Meanwhile, overexpression of circ_CELF1 reduced the sensitivity of NSCLC tumors to anti-PD-1. Therefore, circ_CELF1/miR-491-5p/EGFR axis involved in NSCLC tumor procession, which would provide a new therapeutic target for NSCLC.

## MATERIALS AND METHODS

### Primary samples

Primary samples were collected from 37 cases of lung adenocarcinoma surgery (tumor tissues and adjacent normal tissues) at our hospital. Fresh tissues were immediately put in −80°C. The collection and preservation process are in accordance with the principle of aseptic operation and a complete collection of the patients’ information including sex, age, tumor volume, lymph node metastasis and clinical data, etc. The patients were separated into circ_CELF1 high and low group by medium value of circ_CELF1 expression. A follow-up survival analysis was conducted to determine the correlation between circ_CELF1 expression and patient prognosis. Clinical information of patients was listed in Table 1. This study has been approved by the Medical Ethics Committee of our hospital.

**Table 1:** Patient information.

**Variables**	**Number**
Sex	
Male	20
Female	17
Age	
<50 years	16
≥50 years	21
Histology	
Adenocarcinoma	37
Lymph node metastasis	
Yes	14
No	23
Total	37

### Cell cultured and treatment

Human A549 and H1975 cell lines, and mouse Lewis lung cancer (LLC) and LAA795 cell lines were purchased from the Science Cell Laboratory. Cell lines were cultured in RPMI 1640 with 10% FBS and 100 μL/mL penicillin and streptomycin (Beyotime, China) and placed at 37°C with 5% CO_2_.

### Cell transfection

The overexpressing plasmid of circ_CELF1, miR-491-5p mimics and inhibitors, and the negative controls (NC) were purchased from Gene Pharma (China). Cells transfection was conducted by using Lipofectamine 2000 reagent (Invitrogen, USA) in line with manufacturer’s protocol. In brief, cells were planted in 6-well plates, and a mixture of Lipofectamine 2000 (5 μL) and oligonucleotides (0.75 μg) was added to hatch for 8 hours. After that, the medium was replaced by fresh completed culture medium to incubate for another 24 hours. Cells were then collected for further experiments.

### Wound healing assay

The migration ability of cells was assessed by measuring the movement of cells into a scraped area created by a sterile 200 μl pipette tip. After scratched, cells were washed twice and cultured in media supplemented with 0.1% FBS to eliminate the effect of cell proliferation. The spread of wound closure was photographed at 0 and 48 hours under ×5 objective lens.

### Quantitative Real-time PCR (qRT-PCR)

The frozen fresh tissue or cells were taken and total RNA was extracted according to the operating instructions of the Rneasy Mini Kit. Total RNA concentration was determined by spectrophotometer. Then according to the operating instructions of the reverse transcription kit, the extracted RNA was reverse-transcribed into cDNA and stored at −80°C for later experiments. Quantitative PCR reaction system was configured according to the instructions. The reaction conditions were as follows: 9°C 15 min, 40 cycles, 95°C 15 s in each cycle, 55°C 30 s, 72°C 1 s, 40°C 1 min. The data were calculated by 2^−ΔΔCt^. PCR primers were shown as follows: GAPDH forward primers 5′-GTCAACGGATTTGGTCTGTATT-3′ and reverse primers 5′-AGTCTTCTGGGTGGCAGTGAT-3′.

### Western blot

The protocol in detail have been described previously. Briefly, 50–80 μg protein collected from tissues or cells with RIPA lysis buffer was loaded via SDS-PAGE and transferred onto nitrocellulose membranes (Absin Bioscience Inc., China). The membrane was firstly incubated in 5% non-fat milk blocking buffer for horizontal mode for 1 h. Subsequently, the membrane was incubated with primary antibodies overnight at 4°C and incubated with secondary antibodies for 1 h at room temperature. Eventually, the membranes were scanned using an Odyssey, and the intensity of the protein band was analyzed with Odyssey software (LI-COR, USA).

### CCK8 assay

Cells were cultured in 96-well cell plates and added CCK-8 buffer (MedChemExpress, USA) at 0, 24, 48, and 72 h. 2 h later, measure 450 OD value with an MK3ELISA photometer (Tecan, Germany).

### Tumor xenograft transplantation assay

Nude mice were purchased from the Beijing Charles river. Male BALB/c nude mice aged 4–6 weeks were maintained. All mice were randomized, and the investigators were blinded to the group assignment. We resuspended 106 stable circ_CELFl expressing A549 cells (per mouse) in 100 μl of PBS and injected them into the lateral tail vein. The mice were sacrificed after 30 days; the lungs were resected, embedded in paraffin, and stained with hematoxylin and eosin (H&E); and lung metastases were counted. The research protocol was approved by the Animal Care and Use Committee of our hospital.

### Mice xenograft anti-PD-1 therapy study

The experiments in the C57BL/6 mice were approved by the Animal Experimentation Ethics Committee of our hospital. A total of 2 × 10^6^ cells (LLC with or without increased circ_CELF1expression) were implanted subcutaneously in the left flank of the six- week-old C57BL/6 mice. When tumors reached a size of approximately 100 mm^3^, the mice were randomly assigned to 4 groups. Then, the mice were injected in the tail vein with a PD-1 monoclonal IgG antibody or its mouse isotype control at 100 μg per dose three times a week for two weeks. Animals were euthanized when tumors reached a maximum of 1000 mm^3^. The day that the mice received the first therapy is considered day 1.

### Cell cycle analysis

NSCLC cells were washed with pre-cold PBS and subsequently fixed with 70% ethanol overnight. Following digestion by RNase, the cell medium was filtered with a 300-mesh sieve, centrifuged at 1000 RPM at 4°C for 5 min. The cell pellets were stained with 1ml PI solution and analyzed by flow cytometry.

### Luciferase reporter assay

The potential binding sequences between miR-491-5p and circ_CELF1 or EGFR were determined by ENCORI website. The wild-type (WT) or mutant (MUT) circ_CELF1 or 3′UTR of EGFR was amplified and cloned into pmirGLO vector separately. 20 mmol/L miR-RNA mimic or miR-NC and circ_CELF1-WT/circ_CELF1-mutation or EGFR-WT/EGFR-mutation were co-transfected into HEK293T cells. Luciferase activity was measured with Luciferase Reporter Assay Kit (Biovision, China) on luminometer (Berthold, Germany) 48 hrs post-transfection.

### RNA binding protein immunoprecipitation (RIP) with AGO2

AGO2 is a mediator of pre-miRNA cleavage. The immunoprecipitation of AGO2 could enrich the miRNA-mRNA complex. Similarly, the circRNA-miRNA complex could also be precipitated by AGO2 antibody. Therefore, RIP with AGO2 was adopted to evaluate the direct interaction between circRNA and miRNA. RIP with AGO2 was conducted using a Magna RIP RNA-binding protein immunoprecipitation kit (Millipore, USA) in accordance with manufacturer’s instructions. Briefly, antibodies for AGO2 or IgG were applied to hatch with the cell lysates, followed by centrifuge and qRT-PCR analysis of enrichment of targeted circRNA and miRNA.

### Cell invasion assays

For invasion assay, the chilled Matrigel was diluted with serum-free medium at 1:6 and 100 μL was applied to the transwell chamber, and incubated for 4 hours. The cells were resuspended in serum-free medium. 1 × 10^4^ cells in 100 μL serum-free medium were seeded into the upper chamber. Five hundred microliters completed medium was added to the bottom wells to stimulate migration or invasion. After incubation, the cells were fixed with 4% paraformaldehyde for 15 minutes at room temperature and stained with 0.1% crystal violet for 5 minutes. The cells were observed, imaged, and counted under a microscope.

### RNA stability evaluation

Total RNA and genome DNA were isolated from cells, and separated to two equal parts, followed by treatment with RNase (1 U/mg) at 37°C for 20 minutes. The control group was treated with DEPC water. The levels of linear and circular CELF1 were determined by agarose gel electrophoresis. To determine the half-life of RNA, total RNA was processed by actinomycin D (2 mg/mL) (Sigma, USA), and subjected to qRT-PCR assay.

### Fluorescence *in situ* hybridization (FISH) assay

The localization of circ_CELF1 was determined by using a FISH kit (Sigma, USA) following manufacturer’s description. In brief, Cy3 labelled circ_CELF1, 18s, and U6 were synthesized by Gene Pharma (China). Cells were fixed with 4% PFA and hybridized with probes for 10 hours at 37°C, the nuclei were then stained with DAPI for 10 minutes. The images were photographed by a fluorescence confocal microscope (Carl Zeiss, Germany).

### Statistical analysis

All of the data are analyzed by GraphPad 8.0, calculated as the mean ± SEM and measured by Student’s *t*-test and ANOVA. A two-tailed value of *P* < 0.05 is indicated as statistically significant difference.

## Supplementary Materials

Supplementary Figures

## References

[1] Patz EF Jr, Greco E, Gatsonis C, Pinsky P, Kramer BS, Aberle DR. Lung cancer incidence and mortality in National Lung Screening Trial participants who underwent low-dose CT prevalence screening: a retrospective cohort analysis of a randomised, multicentre, diagnostic screening trial. Lancet Oncol. 2016; 17:590–99. 10.1016/s1470-2045(15)00621-x 27009070 PMC5094059

[2] Iams WT, Porter J, Horn L. Immunotherapeutic approaches for small-cell lung cancer. Nat Rev Clin Oncol. 2020; 17:300–12. 10.1038/s41571-019-0316-z 32055013 PMC7212527

[3] Skoulidis F, Heymach JV. Co-occurring genomic alterations in non-small-cell lung cancer biology and therapy. Nat Rev Cancer. 2019; 19:495–509. 10.1038/s41568-019-0179-8 31406302 PMC7043073

[4] Howlader N, Forjaz G, Mooradian MJ, Meza R, Kong CY, Cronin KA, Mariotto AB, Lowy DR, Feuer EJ. The Effect of Advances in Lung-Cancer Treatment on Population Mortality. N Engl J Med. 2020; 383:640–49. 10.1056/NEJMoa1916623 32786189 PMC8577315

[5] Szabo L, Salzman J. Detecting circular RNAs: bioinformatic and experimental challenges. Nat Rev Genet. 2016; 17:679–92. 10.1038/nrg.2016.114 27739534 PMC5565156

[6] Kristensen LS, Andersen MS, Stagsted LVW, Ebbesen KK, Hansen TB, Kjems J. The biogenesis, biology and characterization of circular RNAs. Nat Rev Genet. 2019; 20:675–91. 10.1038/s41576-019-0158-7 31395983

[7] Beermann J, Piccoli MT, Viereck J, Thum T. Non-coding RNAs in Development and Disease: Background, Mechanisms, and Therapeutic Approaches. Physiol Rev. 2016; 96:1297–325. 10.1152/physrev.00041.2015 27535639

[8] Xiao-Long M, Kun-Peng Z, Chun-Lin Z. Circular RNA circ_HIPK3 is down-regulated and suppresses cell proliferation, migration and invasion in osteosarcoma. J Cancer. 2018; 9:1856–62. 10.7150/jca.24619 29805712 PMC5968774

[9] Lu C, Fu L, Qian X, Dou L, Cang S. Knockdown of circular RNA circ-FARSA restricts colorectal cancer cell growth through regulation of miR-330-5p/LASP1 axis. Arch Biochem Biophys. 2020; 689:108434. 10.1016/j.abb.2020.108434 32473899

[10] Zhang PF, Pei X, Li KS, Jin LN, Wang F, Wu J, Zhang XM. Circular RNA circFGFR1 promotes progression and anti-PD-1 resistance by sponging miR-381-3p in non-small cell lung cancer cells. Mol Cancer. 2019; 18:179. 10.1186/s12943-019-1111-2 31815619 PMC6900862

[11] Vlasova-St Louis I, Bohjanen PR. Coordinate regulation of mRNA decay networks by GU-rich elements and CELF1. Curr Opin Genet Dev. 2011; 21:444–51. 10.1016/j.gde.2011.03.002 21497082 PMC3146975

[12] Xia L, Sun C, Li Q, Feng F, Qiao E, Jiang L, Wu B, Ge M. CELF1 is Up-Regulated in Glioma and Promotes Glioma Cell Proliferation by Suppression of CDKN1B. Int J Biol Sci. 2015; 11:1314–24. 10.7150/ijbs.11344 26535026 PMC4625542

[13] House RP, Talwar S, Hazard ES, Hill EG, Palanisamy V. RNA-binding protein CELF1 promotes tumor growth and alters gene expression in oral squamous cell carcinoma. Oncotarget. 2015; 6:43620–34. 10.18632/oncotarget.6204 26498364 PMC4791255

[14] Torigoe H, Yamamoto H, Sakaguchi M, Youyi C, Namba K, Sato H, Shien K, Soh J, Suzawa K, Tomida S, Tsukuda K, Miyoshi S, Toyooka S. Tumor-suppressive effect of LRIG1, a negative regulator of ErbB, in non-small cell lung cancer harboring mutant EGFR. Carcinogenesis. 2018; 39:719–27. 10.1093/carcin/bgy044 29546323

[15] Roskoski R Jr. Small molecule inhibitors targeting the EGFR/ErbB family of protein-tyrosine kinases in human cancers. Pharmacol Res. 2019; 139:395–411. 10.1016/j.phrs.2018.11.014 30500458

[16] Zheng L, Wang Y, Xu Z, Yang Q, Zhu G, Liao XY, Chen X, Zhu B, Duan Y, Sun J. Concurrent EGFR-TKI and Thoracic Radiotherapy as First-Line Treatment for Stage IV Non-Small Cell Lung Cancer Harboring EGFR Active Mutations. Oncologist. 2019; 24:1031–e612. 10.1634/theoncologist.2019-0285 31040256 PMC6693693

[17] Vo JN, Cieslik M, Zhang Y, Shukla S, Xiao L, Zhang Y, Wu YM, Dhanasekaran SM, Engelke CG, Cao X, Robinson DR, Nesvizhskii AI, Chinnaiyan AM. The Landscape of Circular RNA in Cancer. Cell. 2019; 176:869–81.e13. 10.1016/j.cell.2018.12.021 30735636 PMC6601354

[18] Zhou X, Li J, Zhou Y, Yang Z, Yang H, Li D, Zhang J, Zhang Y, Xu N, Huang Y, Jiang L. Down-regulated ciRS-7/up-regulated miR-7 axis aggravated cartilage degradation and autophagy defection by PI3K/AKT/mTOR activation mediated by IL-17A in osteoarthritis. Aging (Albany NY). 2020; 12:20163–83. 10.18632/aging.103731 33099538 PMC7655186

[19] Li X, Zhang Z, Jiang H, Li Q, Wang R, Pan H, Niu Y, Liu F, Gu H, Fan X, Gao J. Circular RNA circPVT1 Promotes Proliferation and Invasion Through Sponging miR-125b and Activating E2F2 Signaling in Non-Small Cell Lung Cancer. Cell Physiol Biochem. 2018; 51:2324–40. 10.1159/000495876 30537738

[20] Liu Z, Guo S, Sun H, Bai Y, Song Z, Liu X. Circular RNA CircHIPK3 Elevates CCND2 Expression and Promotes Cell Proliferation and Invasion Through miR-124 in Glioma. Front Genet. 2020; 11:1013. 10.3389/fgene.2020.01013 33005182 PMC7485042

[21] Medjebar S, Truntzer C, Perrichet A, Limagne E, Fumet JD, Richard C, Elkrief A, Routy B, Rébé C, Ghiringhelli F. Angiotensin-converting enzyme (ACE) inhibitor prescription affects non-small-cell lung cancer (NSCLC) patients response to PD-1/PD-L1 immune checkpoint blockers. Oncoimmunology. 2020; 9:1836766. 10.1080/2162402X.2020.1836766 33178495 PMC7595630

[22] Juliá EP, Mandó P, Rizzo MM, Cueto GR, Tsou F, Luca R, Pupareli C, Bravo AI, Astorino W, Mordoh J, Martín C, Levy EM. Peripheral changes in immune cell populations and soluble mediators after anti-PD-1 therapy in non-small cell lung cancer and renal cell carcinoma patients. Cancer Immunol Immunother. 2019; 68:1585–96. 10.1007/s00262-019-02391-z 31515670 PMC11028077

[23] Shibaki R, Murakami S, Shinno Y, Matsumoto Y, Yoshida T, Goto Y, Kanda S, Horinouchi H, Fujiwara Y, Yamamoto N, Yamamoto N, Ohe Y. Predictive value of serum VEGF levels for elderly patients or for patients with poor performance status receiving anti-PD-1 antibody therapy for advanced non-small-cell lung cancer. Cancer Immunol Immunother. 2020; 69:1229–36. 10.1007/s00262-020-02539-2 32152703 PMC11027660

