# Supplementary material for: Circular RNA CELF1 drives immunosuppression and anti-PD1 therapy resistance in non-small cell lung cancer via the miR-491-5p/EGFR axis
Source: Aging (Albany NY). 2021 Nov 17;13(22):24560–79. doi: 10.18632/aging.203576 (PMC8660608; doi:10.18632/aging.203576)
Supplement: Supplementary Figures [file aging-13-203576-s001.pdf]

SUPPLEMENTARY FIGURES

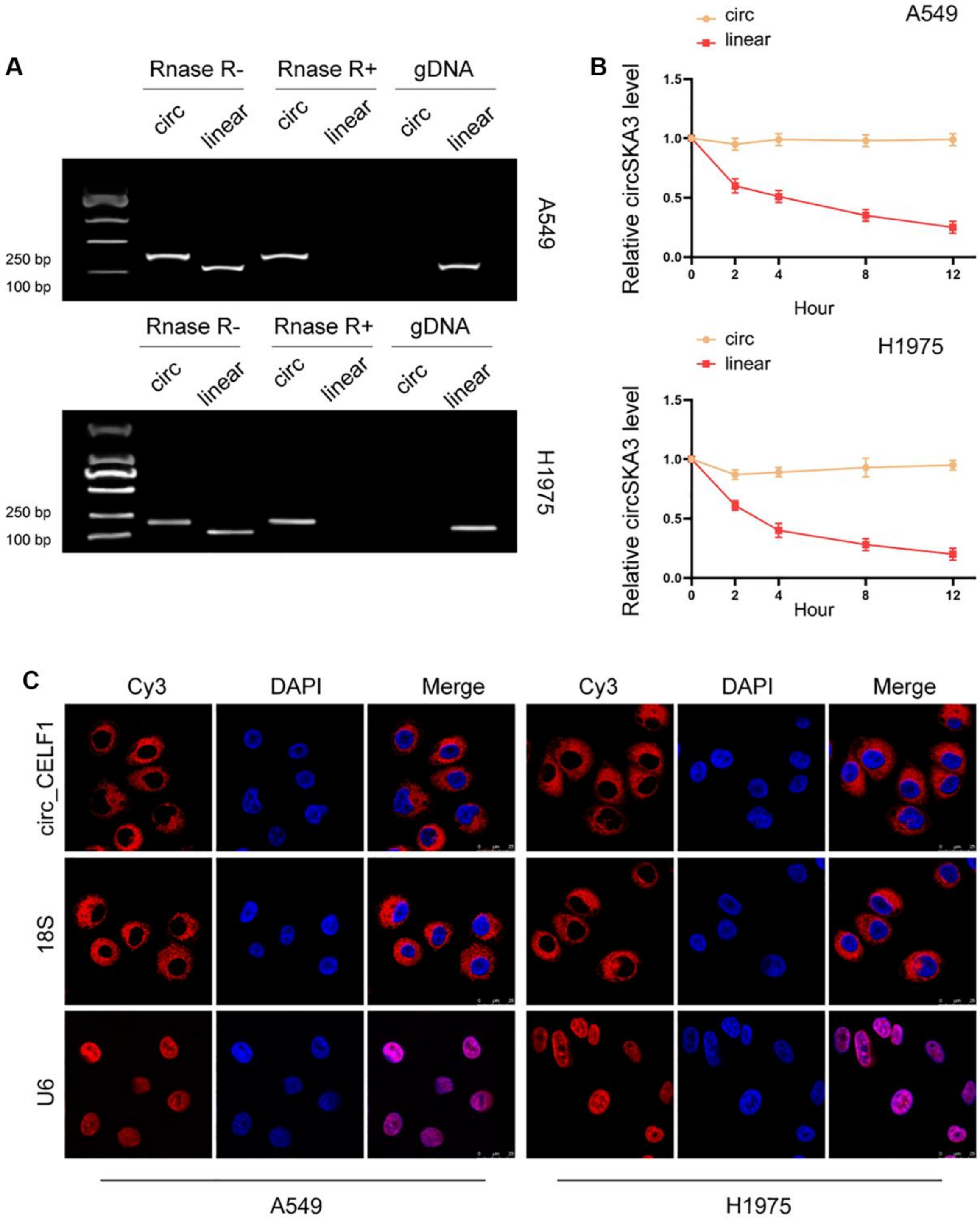

Supplementary Figure 1. Identification of circ_CELF1. (**A**) The circular and linear form of CELF1 expression was measured by agarose gel electrophoresis assays and qRT-PCR in A549 and H1975 cells in the presence or absence of Rnase R treatment. (**B**, **C**) RNA level of circ_CELF1 under actinomycin D treatment. $n = 5$, $^*P < 0.05$.

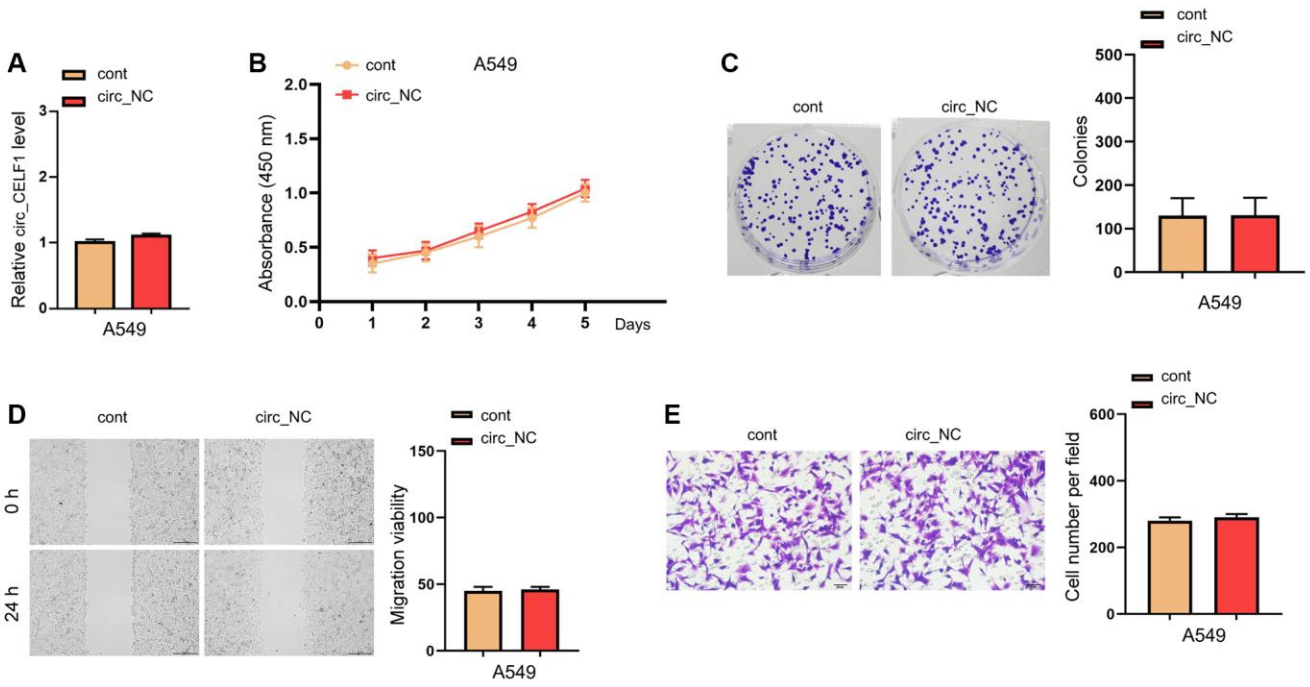

Supplementary Figure 2. Transfection of negative control circRNA do not induces the progression of NSCLC cells *in vitro*. (**A**) The qRT-PCR was performed to confirm the level of circ_CELF1 in NSCLC cells. n = 5, $^{*}P < 0.05$. (**B**) CCK-8 assay was performed to detect the cell viability on A549 cells. n = 6, $^{*}P < 0.05$. (**C**) The clone formation assay on A549 cells. n = 3, $^{*}P < 0.05$. (**D**) The wound healing assay was performed on A549 cells. n = 3, $^{*}P < 0.05$. (**E**) The ability of invasion was explored on A549 cells by Transwell. n = 4, $^{*}P < 0.05$.

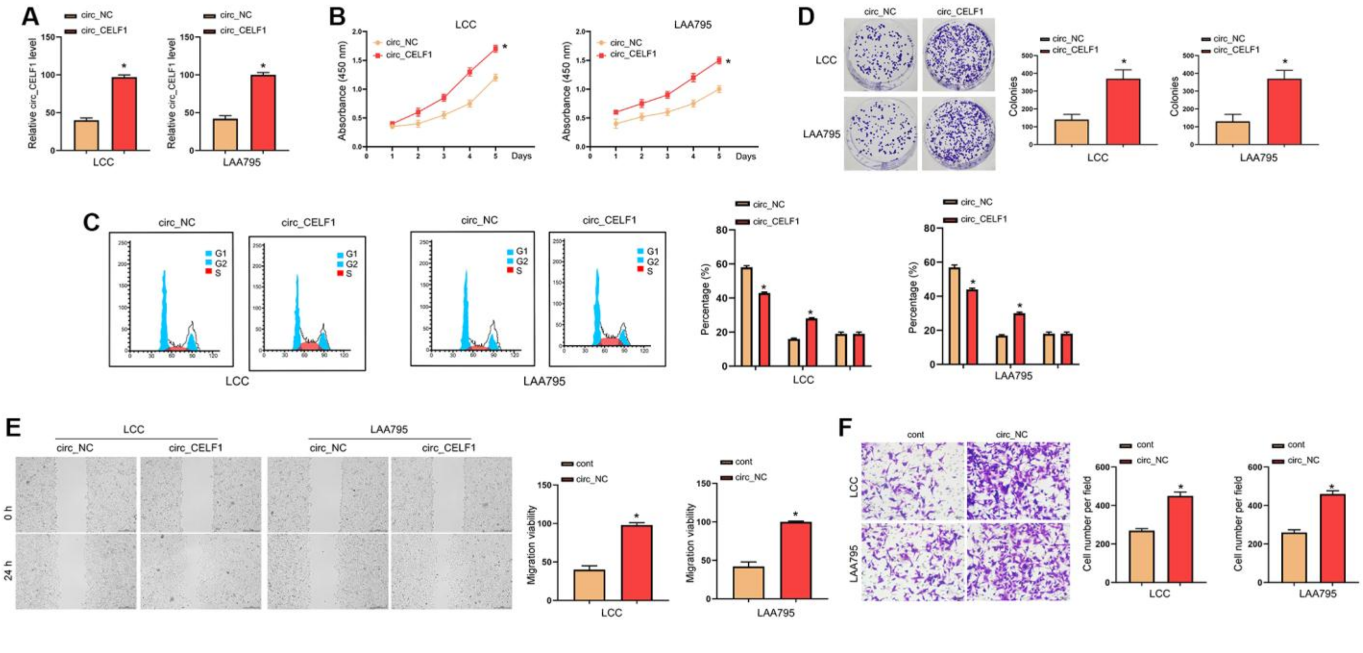

Supplementary Figure 3. Forced expression of circ_CELF1 induces the progression of LCC and LAA795 cells *in vitro*. (**A**) The qRT-PCR was performed to confirm the transfection efficiency of circ_CELF1 in LCC and LAA795 cells. $^{*}P < 0.05$. (**B**) CCK-8 assay was performed to detect the cell viability on LCC and LAA795 cells. n = 6, $^{*}P < 0.05$. (**C**) The cell cycle was analyzed on LCC and LAA795 cells. n = 3, $^{*}P < 0.05$. (**D**) The clone formation assay on LCC and LAA795 cells. n = 3, $^{*}P < 0.05$. (**E**) The wound healing assay was performed on LCC and LAA795 cells. n = 3, $^{*}P < 0.05$. (**F**) The ability of invasion was explored on LCC and LAA795 cells by Transwell. n = 4, $^{*}P < 0.05$.

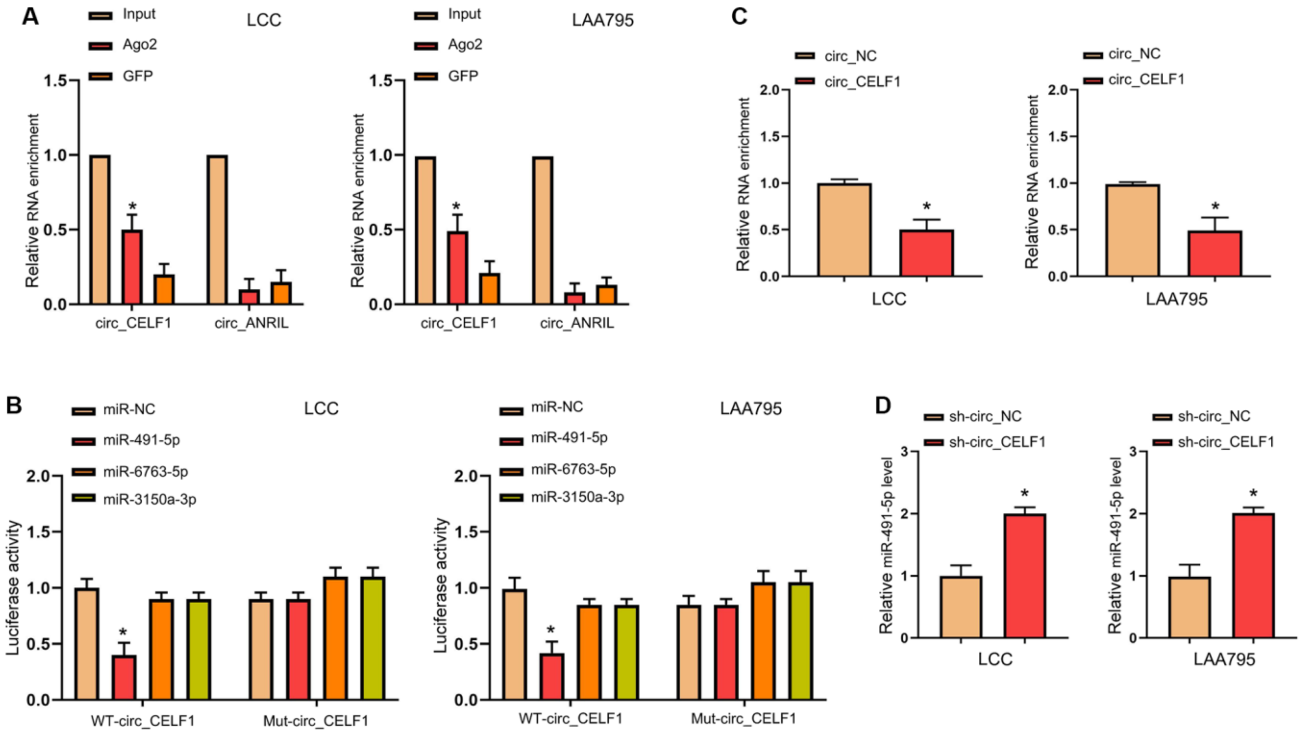

Supplementary Figure 4. circ_CELF1 binds miR-491-5p in LCC and LAA795 cells. (**A**) AGO2 RIP experiments were performed using an antibody against Ago2 on extracts from LCC and LAA795 cells. $n = 3$, $^{*}P < 0.05$. (**B**) The luciferase activity of WT- circ_CELF1 or mutant circ_CELF1 in LCC and LAA795 cells after co-transfection with miR-491-5p, miR-6763-5p, and miR-3150a-3p. $n = 3$, $^{*}P < 0.05$. (**C–D**) The expression of miR-491-5p in LCC and LAA795 cells was explored by RT-PCR under downregulation of circ_CELF1. $n = 4$, $^{*}P < 0.05$.

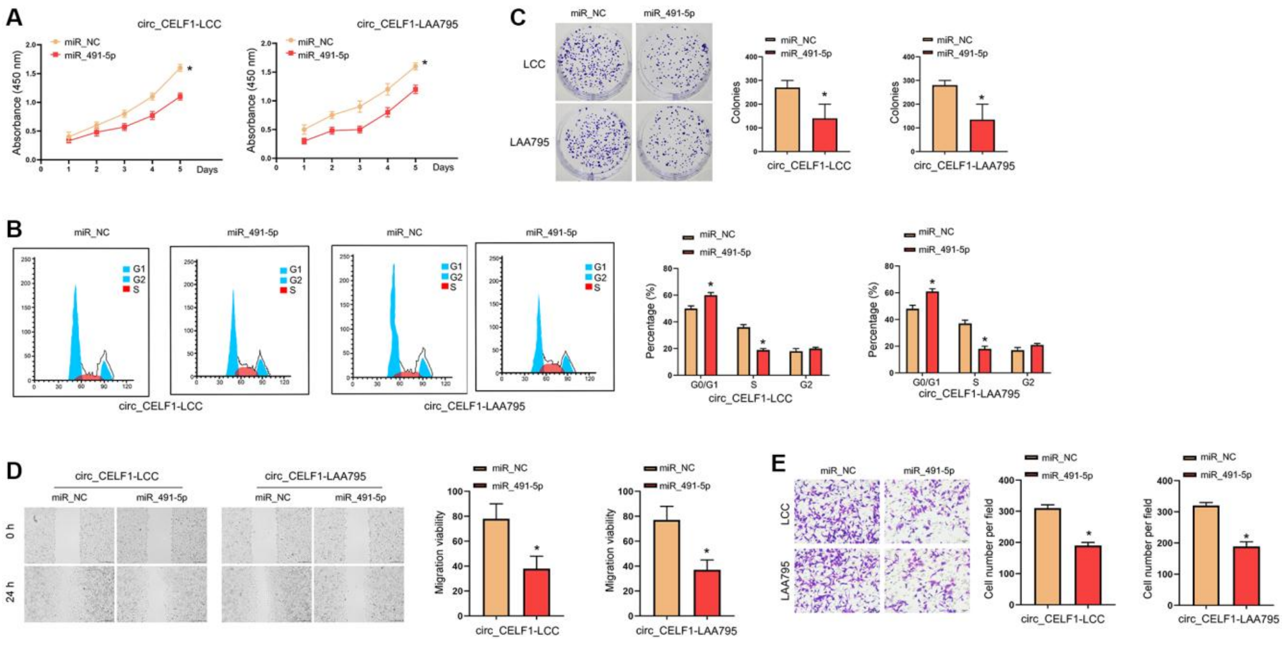

Supplementary Figure 5. Forced expression of miR-491-5p remits the cell progression in stabled circ_CELF1 expression LCC and LAA795 cells. (**A**) CCK-8 assay was performed to detect the effect of miR-491-5p on overexpression of circ_CELF1 LCC and LAA795 cells. $n = 6$, $^{*}P < 0.05$. (**B**) The cell cycle was explored on overexpression of circ_CELF1 LCC and LAA795 cells after miR-491-5p transfection by flow cytometry. $n = 6$, $^{*}P < 0.05$. (**C**) The clone formation assay was performed on overexpression of circ_CELF1 LCC and LAA795 cells. $n = 5$, $^{*}P < 0.05$. (**D**) Wound healing assay was used to confirm the migration ability. $n = 5$, $^{*}P < 0.05$. (**E**) The migration and invasion ability was explored by Transwell. $n = 6$, $^{*}P < 0.05$, $^{**}P < 0.01$.

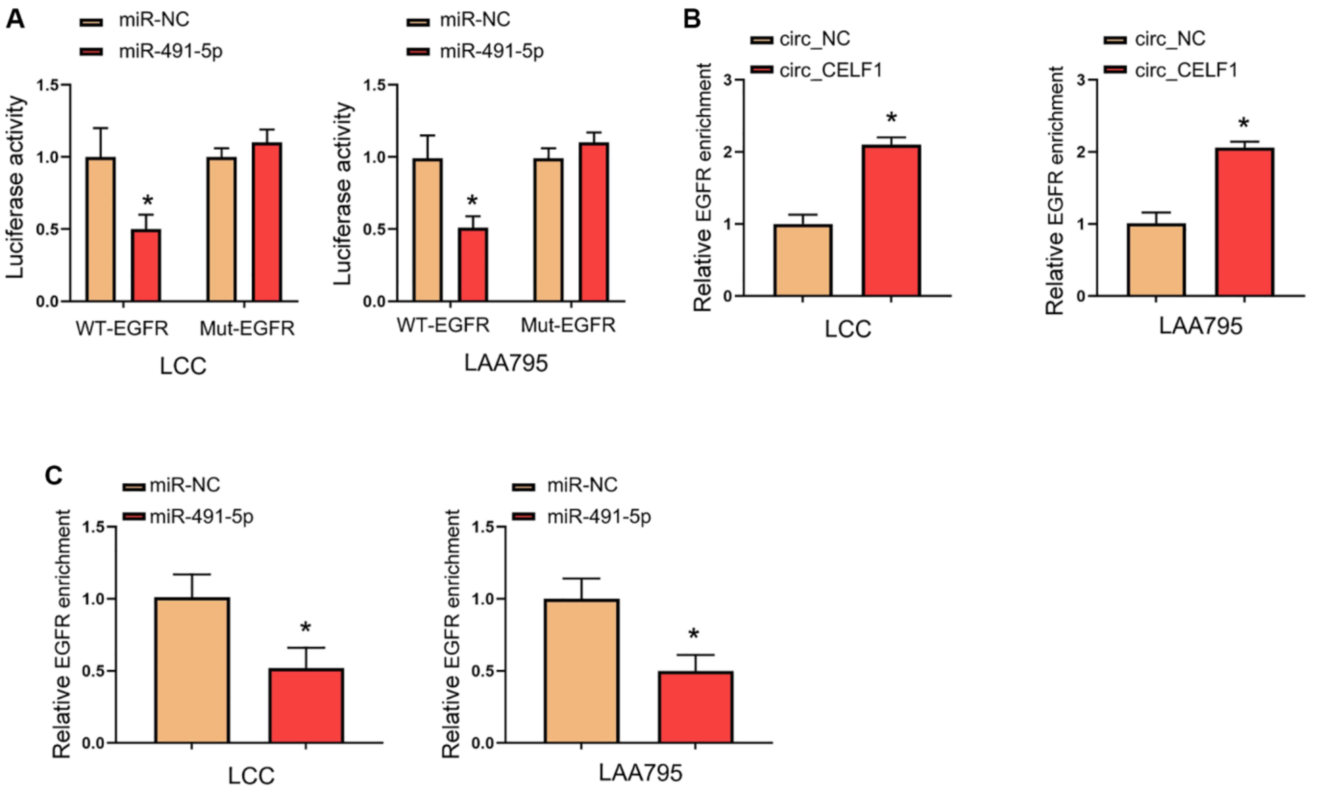

Supplementary Figure 6. EGFR is a target of miR-491-5p in LCC and LAA795 cells. (**A**) Luciferase assay confirmed the relationship between EGFR and miR-491-5p (lower). n = 3, $^{*}P < 0.05$. (**B**, **C**) The expression of EGFR was detected in LCC and LAA795 cells after transfection with circ_CELF1 or miR-491-5p was detected by RT-PCR assay. n = 3, $^{*}P < 0.05$.

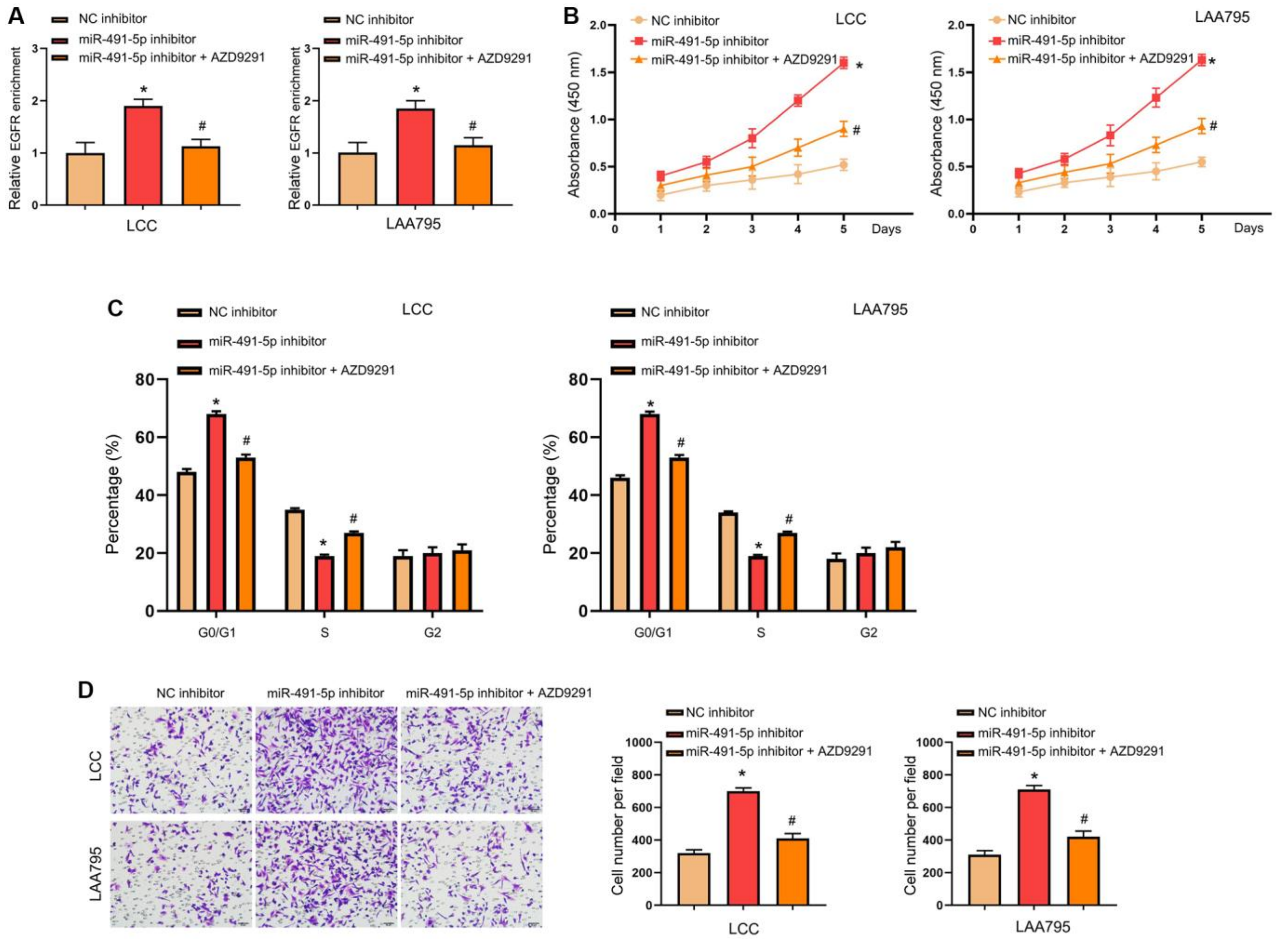

Supplementary Figure 7. EGFR blockage prevents LCC and LAA795 cell progression induced by miR-491-5p inhibition. (**A**) The expression level of EGFR was confirmed by RT-PCR assay. (**B**) CCK-8 assay was performed to detect cell viability in LCC and LAA795 cells. (**C**) The cell cycle was explored in LCC and LAA795 cells after miR-491-5p inhibitor transfection and AZD-9291 treatment by flow cytometry. (**D**) The invasion ability was explored by Transwell. $n = 4$, $^{*}P < 0.05$ vs. NC inhibitor, $^{\#}P < 0.05$ vs. miR-491-5p inhibitor.

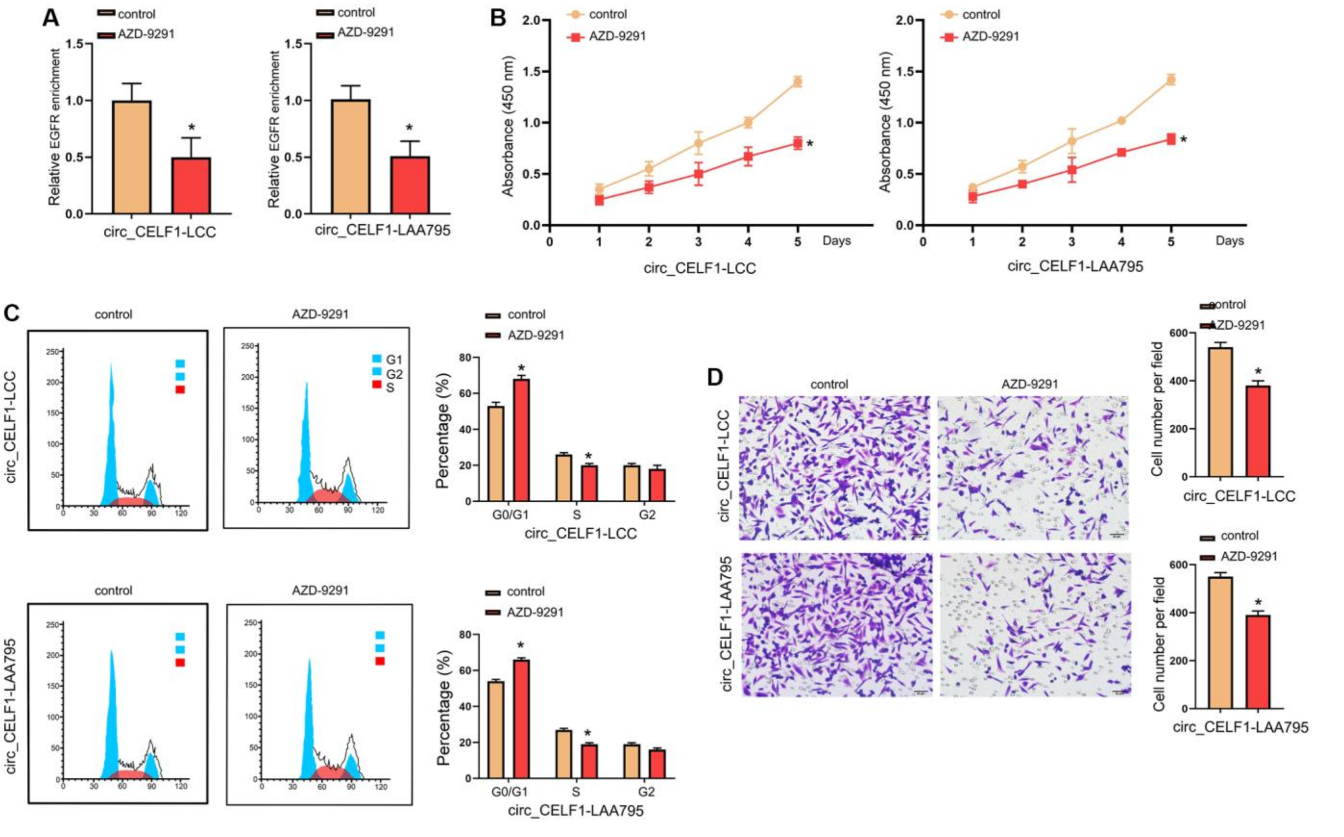

Supplementary Figure 8. Inhibition of EGFR prevents cell progression in stabled circ_CELF1 expressed LCC and LAA795 cells. (**A**) The knockdown efficiency of AZD-9291 was confirmed by qRT-PCR. $n = 4$, $^{*}P < 0.05$. (**B**) CCK-8 assay was performed to detect the effect of MK-1775 in NSCLC cells. $n = 4$, $^{*}P < 0.05$. (**C**) The cell cycle was explored in NSCLC cells after miR-491-5p transfection by flow cytometry. $n = 4$, $^{*}P < 0.05$. (**D**) The invasion ability was explored by Transwell. $n = 4$, $^{*}P < 0.05$.

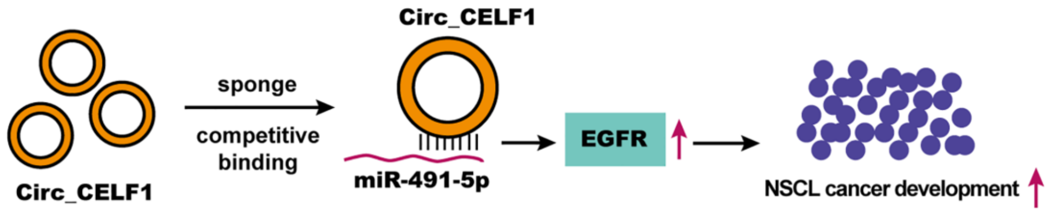

Supplementary Figure 9. Schematic illustration of circ_CELF1/miR-491-5p/EGFR regulatory network in NSCLC cells.
